# Gene expression polymorphism underpins evasion of host immunity in an asexual lineage of the Irish potato famine pathogen

**DOI:** 10.1186/s12862-018-1201-6

**Published:** 2018-07-05

**Authors:** Marina Pais, Kentaro Yoshida, Artemis Giannakopoulou, Mathieu A. Pel, Liliana M. Cano, Ricardo F. Oliva, Kamil Witek, Hannele Lindqvist-Kreuze, Vivianne G. A. A. Vleeshouwers, Sophien Kamoun

**Affiliations:** 1 The Sainsbury Laboratory, Norwich Research Park, Colney Ln, Norwich, NR4 7UH UK; 2 Graduate School of Agricultural Science, Kobe University, Kobe, Japan; 3 National Hellenic Research Foundation, Institute of Biology, Medicinal Chemistry & Biotechnology, Athens, Greece; 4 Wageningen UR Plant Breeding, Wageningen, The Netherlands; 5 Institute of Food and Agricultural Sciences, Department of Plant Pathology, Indian River Research and Education Center, University of Florida, Fort Pierce, FL USA; 6 Genetics and Biotechnology Division, International Rice Research Institute, Los Baños, Philippines; 7 International Potato Center, Lima, Peru

**Keywords:** Asexual reproduction, Clonal lineage, *Phytophthora infestans*, Emergent pathogen, Evolution, Immunity, Phenotypic plasticity, Expression polymorphism, Structural variation, Copy number variation, Loss of heterozygosity

## Abstract

**Background:**

Outbreaks caused by asexual lineages of fungal and oomycete pathogens are a continuing threat to crops, wild animals and natural ecosystems (Fisher MC, Henk DA, Briggs CJ, Brownstein JS, Madoff LC, McCraw SL, Gurr SJ, Nature 484:186–194, 2012; Kupferschmidt K, Science 337:636–638, 2012). However, the mechanisms underlying genome evolution and phenotypic plasticity in asexual eukaryotic microbes remain poorly understood (Seidl MF, Thomma BP, BioEssays 36:335–345, 2014). Ever since the 19th century Irish famine, the oomycete *Phytophthora infestans* has caused recurrent outbreaks on potato and tomato crops that have been primarily caused by the successive rise and migration of pandemic asexual lineages (Goodwin SB, Cohen BA, Fry WE, Proc Natl Acad Sci USA 91:11591–11595, 1994; Yoshida K, Burbano HA, Krause J, Thines M, Weigel D, Kamoun S, PLoS Pathog 10:e1004028, 2014; Yoshida K, Schuenemann VJ, Cano LM, Pais M, Mishra B, Sharma R, Lanz C, Martin FN, Kamoun S, Krause J, et al. eLife 2:e00731, 2013; Cooke DEL, Cano LM, Raffaele S, Bain RA, Cooke LR, Etherington GJ, Deahl KL, Farrer RA, Gilroy EM, Goss EM, et al. PLoS Pathog 8:e1002940, 2012). However, the dynamics of genome evolution within these clonal lineages have not been determined. The objective of this study was to use a comparative genomics and transcriptomics approach to determine the molecular mechanisms that underpin phenotypic variation within a clonal lineage of *P. infestans*.

**Results:**

Here, we reveal patterns of genomic and gene expression variation within a *P. infestans* asexual lineage by comparing strains belonging to the South American EC-1 clone that has dominated Andean populations since the 1990s (Yoshida K, Burbano HA, Krause J, Thines M, Weigel D, Kamoun S, PLoS Pathog 10e1004028, 2014; Yoshida K, Schuenemann VJ, Cano LM, Pais M, Mishra B, Sharma R, Lanz C, Martin FN, Kamoun S, Krause J, et al. eLife 2:e00731, 2013; Delgado RA, Monteros-Altamirano AR, Li Y, Visser RGF, van der Lee TAJ, Vosman B, Plant Pathol 62:1081–1088, 2013; Forbes GA, Escobar XC, Ayala CC, Revelo J, Ordonez ME, Fry BA, Doucett K, Fry WE, Phytopathology 87:375–380, 1997; Oyarzun PJ, Pozo A, Ordonez ME, Doucett K, Forbes GA, Phytopathology 88:265–271, 1998). We detected numerous examples of structural variation, nucleotide polymorphisms and loss of heterozygosity within the EC-1 clone. Remarkably, 17 genes are not expressed in one of the two EC-1 isolates despite apparent absence of sequence polymorphisms. Among these, silencing of an effector gene was associated with evasion of disease resistance conferred by a potato immune receptor.

**Conclusions:**

Our findings highlight the molecular changes underpinning the exceptional genetic and phenotypic plasticity associated with host adaptation in a pandemic clonal lineage of a eukaryotic plant pathogen. We observed that the asexual *P. infestans* lineage EC-1 can exhibit phenotypic plasticity in the absence of apparent genetic mutations resulting in virulence on a potato carrying the *Rpi-vnt1.1* gene. Such variant alleles may be epialleles that arose through epigenetic changes in the underlying genes.

**Electronic supplementary material:**

The online version of this article (10.1186/s12862-018-1201-6) contains supplementary material, which is available to authorized users.

## Background

Sexual reproduction is an important source of genetic diversity yet many asexual fungal and oomycete pathogens can rapidly adapt to their host environment and cause destructive pandemics [1–6]. Filamentous plant pathogens, including the soil-borne wilt fungus *Verticillium dahliae*, the rice and wheat blast fungus *Magnaporthe oryzae*, several rust fungi and *Phytophthora* spp. have widespread clonal populations that are a threat to managed and natural ecosystems as well as global food security [4, 7–14]. The establishment of these asexual populations is favored by agricultural practices such as monoculture and year-round crop production [15]. Therefore, asexual reproduction in plant pathogens is an important feature with serious epidemiological and agronomic implications [16, 17]. However, we know little about genome evolution and phenotypic plasticity in asexual lineages of plant pathogens, and to which degree genetic variation in these lineages affects the virulence profile of pandemic pathogen populations.

*Phytophthora infestans* is a heterothallic oomycete capable of both sexual and asexual reproduction [18, 19]. It causes the late blight disease in several plants in the Solanaceae genus *Solanum* and is renowned for its capacity to rapidly overcome resistant host cultivars [19, 20]. Successive emergence and spread of asexual lineages is a recurrent threat to potato cultivation and one of the main reasons why late blight remains the most destructive disease of potato worldwide [7, 16, 21, 22, 23]. Interestingly, there is evidence that phenotypic variation has emerged within clonal lineages of *P. infestans* with potential consequences on virulence [24, 25–28].

The asexual lineage EC-1 was first detected in 1990 in Ecuador as a dominant genotype with the A1 mating type and IIa mitochondrial haplotype [16, 24, 29]. In Ecuador and neighboring Colombia, EC-1 displaced the pandemic US-1 clone to dominate Andean populations of *P. infestans* associated with cultivated and wild potatoes [16, 24, 29–31]. As part of a population genomic study of ancient and modern *P. infestans* populations, we previously generated whole genome shotgun sequences of two EC-1 isolates, P13527 and P13626, using Illumina paired-end reads (~ 51- and 64-fold coverage, respectively) (Additional file 1: Tables S1, S2, S3) [16]. The objective of this study was to use a comparative genomics and transcriptomics approach to determine the molecular mechanisms that underpin phenotypic variation between members of the EC-1 clonal lineage of *P. infestans*.

## Results

### Genetic polymorphisms within the EC-1 clone

We first estimated levels of presence/absence polymorphisms and copy number variation (CNV) within EC-1. We aligned the sequence reads to the reference genome of *P. infestans* strain T30–4 [32] and calculated average breadth of read coverage and read depth per gene as previously described [7]. 62 and 60 of the 18,155 *P. infestans* genes were missing in the P13527 and P13626 genome sequences compared with T30–4, respectively (Additional file 1: Tables S4 and S5). Of these missing genes, 12 showed presence/absence polymorphisms between the two EC-1 isolates (Additional file 1: Table S6). In addition, 69 genes differed in copy number between the two EC-1 isolates (Additional file 1: Table S7). Interestingly, P13527 has a much higher number of copies than P13626 for the majority of the genes with CNV (61 out of 69, 88%) (Additional file 1: Table S7).

**Fig. 1:**
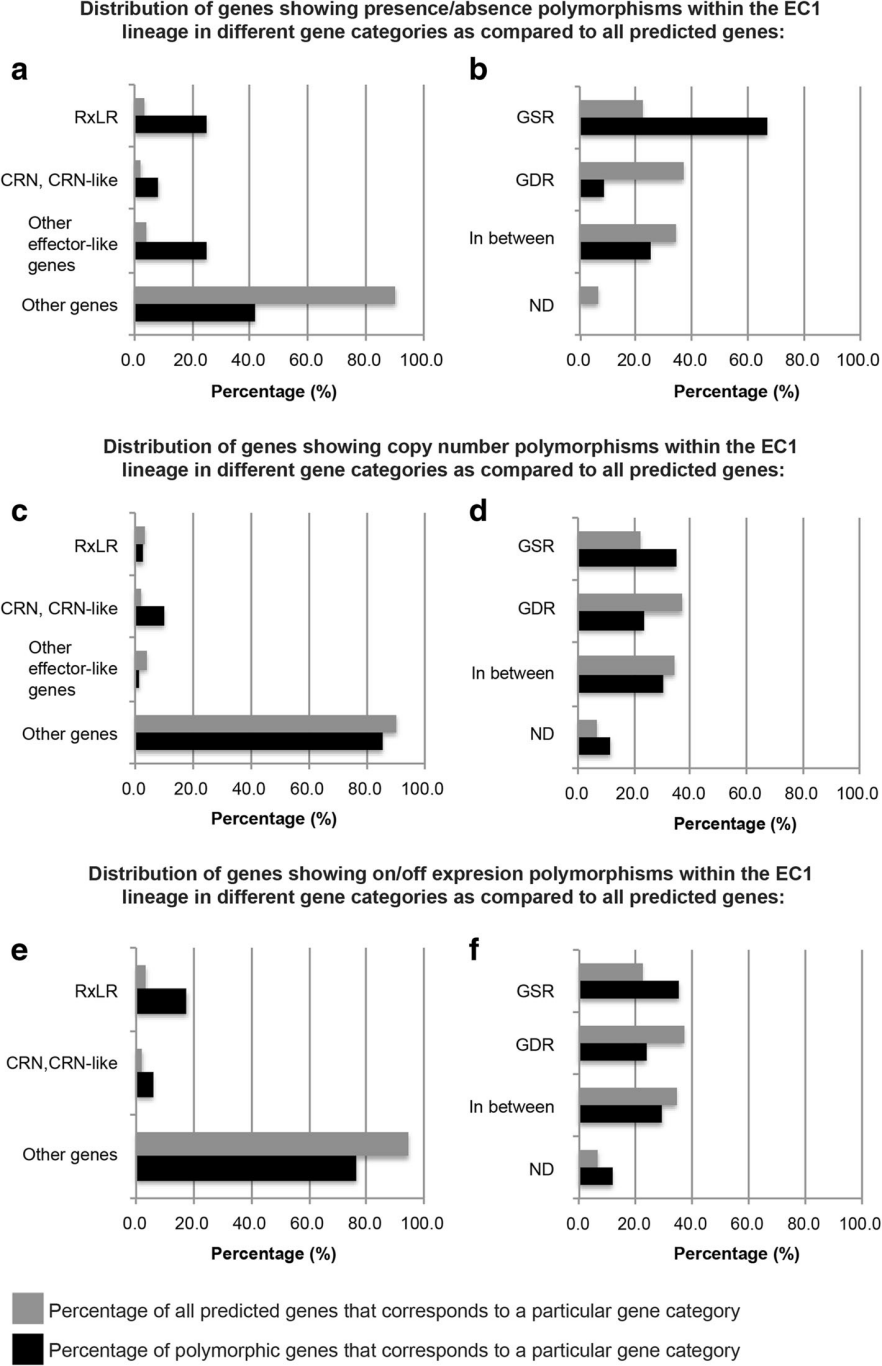
Polymorphisms within the EC-1 lineage. The charts show the distribution of all predicted genes (grey bars) or genes showing polymorphisms within the EC-1 lineage (black bars) among different gene categories. **a** Percentage of RXLR effector genes, Crinkler (CRN) and CRN-like effector genes, other effector-like genes (including genes coding for small cysteine-rich proteins, protein inhibitors, hydrolases, etc.) and non-effector genes in the set of genes showing presence/absence polymorphisms between P13527 and P13626 compared to that in all the predicted genes. Genes encoding disease effectors are over-represented among the genes with presence/absence polymorphisms (one-tailed hypergeometric test, *p* = 3.9 × 10^− 5^). **b** Percentage of genes located in Gene Sparse Regions (GSR), Gene Dense Regions (GDR), in between GSR and GDR, and with location not determined (ND) in the set of genes showing presence/absence polymorphisms between P13527 and P13626 compared to that in all the predicted genes. Genes located in GSR are over-represented among the genes with presence/absence polymorphisms (one-tailed hypergeometric test, *p* = 0.0012). **c** Percentage of RXLR effector genes, Crinkler (CRN) and CRN-like effector genes, other effector-like genes (including genes coding for small cysteine-rich proteins, protein inhibitors, hydrolases, etc.) and non-effector genes in the set of genes showing gene copy number (CN) polymorphisms between P13527 and P13626 compared to that in all the predicted genes. **d** Percentage of genes located in Gene Sparse Regions (GSR), Gene Dense Regions (GDR), in between GSR and GDR, and with location not determined (ND) in the set of genes showing gene copy number (CN) polymorphisms between P13527 and P13626 compared to that in all the predicted genes. Genes located in GSR are over-represented among the genes with CN polymorphisms (one-tailed hypergeometric test, *p* = 0.0112). However, the GSR bias for CN polymorphisms is not significant after Bonferroni correction. **e** Percentage of RXLR effector genes, Crinkler (CRN) and CRN-like effector genes, other effector-like genes (including genes coding for small cysteine-rich proteins, protein inhibitors, hydrolases, etc.) and non-effector genes in the set of genes showing on/off expression polymorphisms between P13527 and P13626 compared to that in all the predicted genes. Genes encoding cytoplasmic effectors of the RXLR and CRN families are over-represented among the genes with on/off expression polymorphisms (one-tailed hypergeometric test, *p* = 0.0129). **f** Percentage of genes located in Gene Sparse Regions (GSR), Gene Dense Regions (GDR), in between GSR and GDR, and with location not determined (ND) in the set of genes showing expression polymorphisms between P13527 and P13626 compared to that in all the predicted genes

The *P. infestans* genome exhibits a discontinuous distribution of gene density with rapidly evolving disease effector genes localized to expanded, repeat-rich and gene-sparse regions (GSR) of the genome, in contrast to core ortholog genes, which occupy repeat-poor and gene-dense regions (GDR) [32, 33]. This “two-speed genome” architecture is thought to favor rapid evolution of the pathogen and facilitate adaptation to an ever-changing host environment [33, 34]. We noted that genes located in GSR were over-represented among the genes with presence/absence polymorphisms (*p* = 0.0012) and CNV (*p* = 0.0112, although the GSR bias for CNV was not significant after Bonferroni correction), unlike other gene categories (Fig. 1b and d, Additional file 2: Figure S1). Genes encoding disease effectors were only over-represented among the genes with presence/absence polymorphisms (*p* = 3.9 × 10^− 5^), unlike other gene categories (Fig. 1b, Additional file 2: Figure S1). Among the 12 genes with presence/absence polymorphisms within EC-1, 8 are located in GSR, and 7 encode effector genes (Additional file 1: Table S6). The missing P13626 genes include 3 genes that have sequence similarity to the RXLR-type *Avr2* effectors, which are detected by immune receptors of the R2 class and, therefore, may reflect adaptation to host immunity [35, 36].

Next, we identified single nucleotide polymorphisms (SNPs) between P13527 and P13626, and compared the SNP frequencies to isolate 06_3928A, which belongs to 13_A2, a distinct clonal lineage of *P. infestans* [7]. The number of homozygous SNPs between the two EC-1 isolates was ~ 54-fold lower than between the EC-1 isolates and 06_3928A, consistent with their expected genetic relationships (Additional file 1: Table S8). A large number of SNPs (> 327,000) occurred in heterozygous state within each of the individual EC-1 isolates (Additional file 1: Table S8). The number of these heterozygous SNPs was ~ 11-fold higher than homozygous SNPs between the EC-1 isolates and 06_3928A, indicating the maintenance of a high level of genetic diversity at the heterozygous state within the EC-1 clonal lineage (Additional file 1: Table S8). About one third (111,144) of the heterozygous SNPs are shared between the EC-1 isolates and 06_3928A, indicating that these sites were already polymorphic prior to the split between the EC-1 and 13_A2 clonal lineages (Additional file 1: Table S9). The frequency of genes with homozygous and heterozygous SNPs was not dependent on genome localization and functional category (Additional file 2: Figure S2).

*Phytophthora* spp. are known to be prone to mitotic loss of heterozygosity (LOH), turning heterozygous genotypes to homozygous in the absence of sexual reproduction [37]. To estimate how many homozygous genotypes in segregating sites between P13527 and P13626 were generated by LOH, we calculated the number of segregating sites where one of the EC-1 isolates has homozygous genotypes and the other EC-1 isolate and the 13_A2 isolate 06_3928A have heterozygous genotypes (Additional file 1: Table S9). By assigning the 13_A2 isolate as an outgroup, heterozygous genotypes were estimated to be the ancestral type and homozygous genotypes were predicted to be the novel type generated by LOH in the population of EC-1 isolates, as the most parsimonious explanation. In total, we identified 140,852 heterozygous SNPs that are shared by the two clonal lineages. Of these, 19,983 (14.2%) are homozygous in P13527 and 9725 (6.9%) homozygous in P13626 indicating significant levels of LOH in EC-1 (Additional file 1: Table S9). LOH frequencies did not particularly vary depending on genome localization and functional category (Additional file 2: Figure S3). However, we identified 72 effector genes with high or moderate LOH nucleotide substitution effects based on the method of [38] (Additional file 1: Tables S10 and S11). Examples include the predicted RXLR effector PITG_22925 in P13527 and Nep1-like protein PITG_22916 in P13626, which became homozygous for an allele with a premature stop codon, as well as predicted RXLR effector PITG_22922 in P13527 and CRN effector PITG_06050 in P13626, which became homozygous for an allele with multiple nonsynonymous substitutions (Additional file 1: Tables S10 and S11).

### Gene expression polymorphisms within the EC-1 clone

*P. infestans* is known to exhibit significant levels of gene expression polymorphisms [7]. Next, we investigated the degree to which the transcriptomes of the two EC-1 isolates diverge. We isolated RNA from potato leaves 2 days after inoculation with isolates P13527 and P13626, and prepared and sequenced RNA-seq libraries using Illumina technology. We aligned the sequencing reads to the *P. infestans* T30–4 transcript sequences and estimated the counts per million (CPM) for each of the 18,155 genes. Gene expression of 13,703 genes was detected in at least one of the two isolates. A comparison of the gene expression between the isolates showed overall similarity (Pearson correlation coefficient = 0.967) (Additional file 2: Figure S4).

In total, 17 genes had no detectable RNA-seq reads in one of the two EC-1 isolates but were over the threshold CPM value in the other (see details in Methods). These genes had no fixed nucleotide differences between the two EC-1 isolates in the upstream (< 5 kbp), the coding and downstream (< 5 kbp) sequences based on alignments to the reference genome (Additional file 1: Table S12) (Additional file 2: Figure S5). However, of the 17 genes, 12 displayed nucleotide polymorphisms in heterozygous states in one of the two EC-1 isolates (Additional file 1: Table S12, Additional file 2: Figure S5). Of the 17 genes, 14 genes were expressed in *P. infestans* isolate 06_3928A, indicating that these genes were most likely silenced in the EC-1 lineage (Additional file 1: Table S12). Although these overall numbers are small, genes encoding cytoplasmic effectors of the RXLR and CRN families were over-represented among the genes with on/off expression polymorphisms (*p* = 0.0129) (Fig. 1e). However, we did not note an enrichment in GSR located genes (Fig. 1f).

**Fig. 2:**
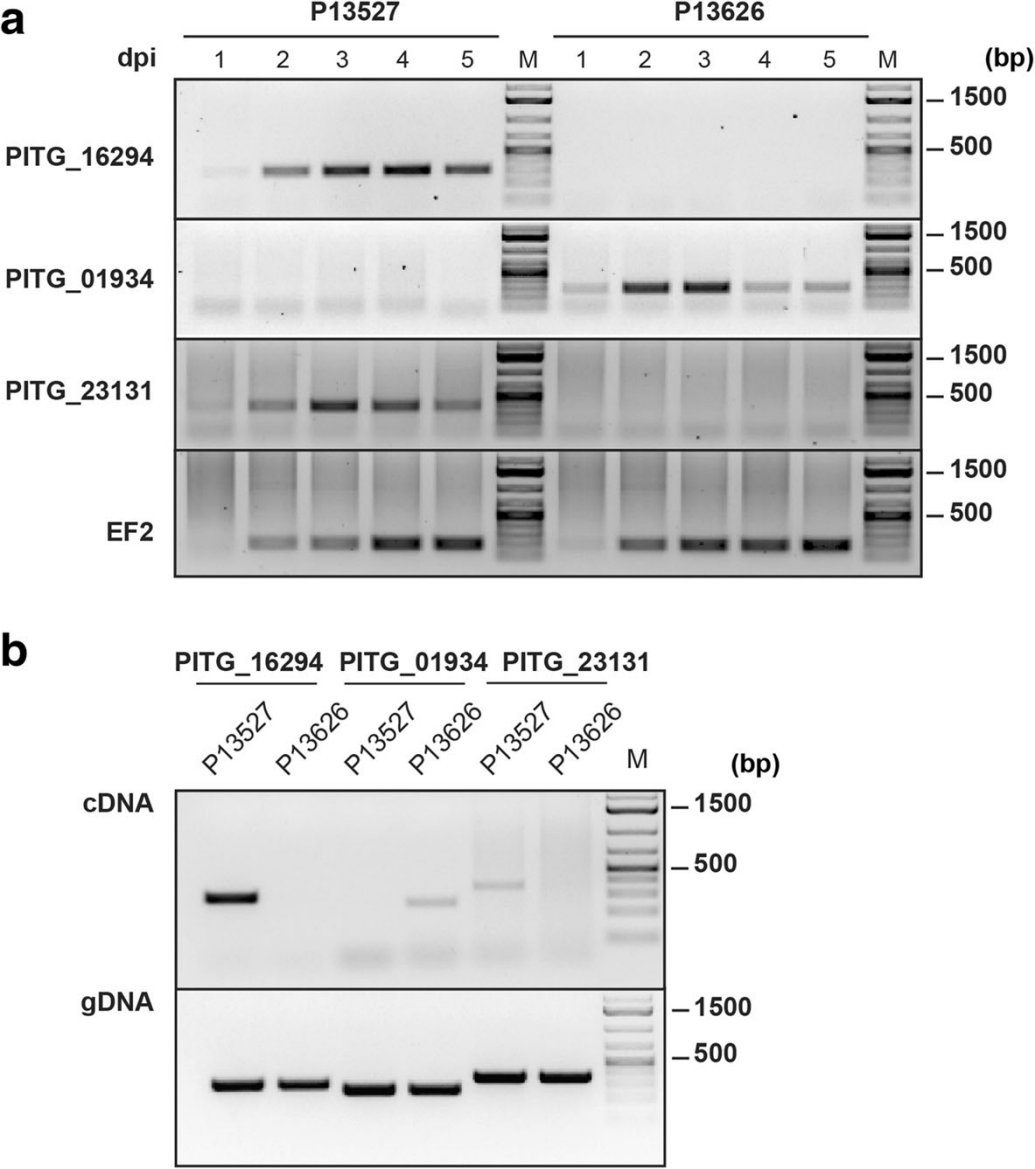
Validation of RXLR effector genes’ expression polymorphisms between P13527 and P13626. **a** RT-PCR experiments performed with cDNA samples from infected potato leaves. Samples were collected from 1 to 5 days post-infection (dpi) with P13527 and P13626. Primers specific for the RXLR effector genes PITG_16294, PITG_01934 and PITG_21131 were used for amplification. Amplification of the *EF2* (*Elongation Factor 2*) cDNA was included as positive control. ‘M’ indicates the lanes where a DNA molecular weight maker was loaded. **b** PCR experiments performed with cDNA (top panel) and gDNA (bottom panel) samples from P13527 and P13626 mycelia. Primers specific for the RXLR effector genes PITG_16294, PITG_01934 and PITG_21131 were used for amplification. ‘M’ indicates the lanes where a DNA molecular weight maker was loaded

Among the genes with on/off expression polymorphisms, three encode RXLR effectors (Additional file 1: Table S12). To validate this finding, we isolated total RNA from potato leaves 1 to 5 days after inoculation with P13527 and P13626, and performed RT-PCR analyses (Fig. 2). We detected transcripts of PITG_16294 and PITG_23131 in P13527 samples but not in P13626 and transcripts of PITG_01934 in P13626 only (Fig. 2). These expression patterns are consistent with the RNA-seq analyses and confirmed that the genes display expression polymorphisms within the EC-1 lineage.

### Loss of expression of the RXLR *Avrvnt1* effector gene is associated with virulence on resistant potatoes

**Fig. 3:**
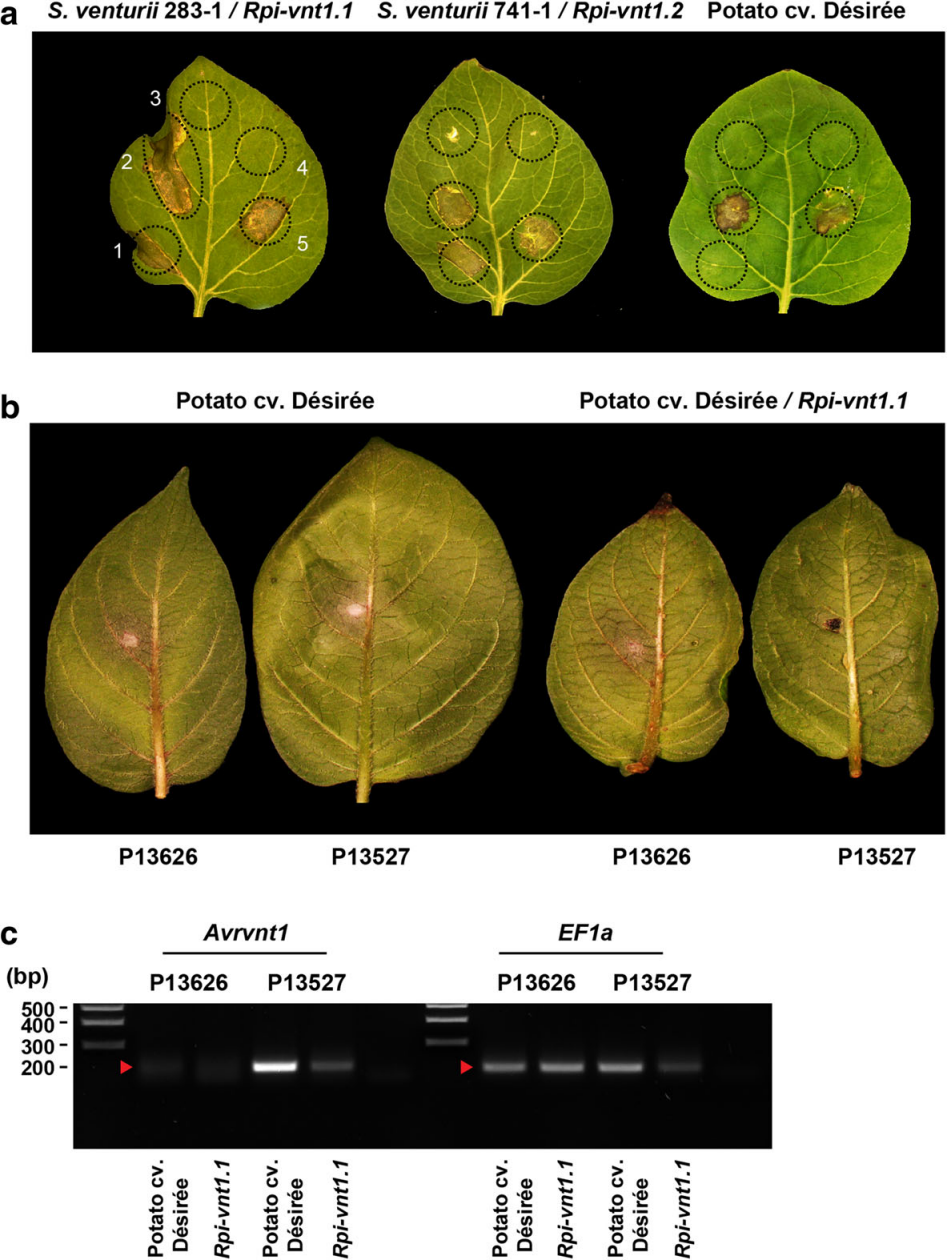
Identification of *Avrvnt1* as the cognate effector for *Rpi-vnt1.* **a** Agroinfiltration of *Avrvnt1* leads to cell death in *Solanum venturii* 283–1 and 741–1 carrying *Rpi-vnt1.1* and *Rpi-vnt1.2*, respectively*,* but not in susceptible potato cultivar Désirée; (1): *Avrvnt1*, (2): *Avrvnt1* and *Rpi-vnt1.1*, (3): *Rpi-vnt1.1*, (4): empty vector pK7WG2 was used as a negative control in this experiment, (5): *R3a* and *Avr3a* were used as a positive control *R/Avr* pair in this experiment. Note that co-expression of *Avrvnt1* and *Rpi-vnt1.1* in potato cultivar Désirée (2, leaf on right side) also results in hypersensitive cell death. **b** Differential virulence of P13626 and P13527 on plants expressing *Rpi-vnt1.1.* **c** RT-PCR experiments performed with cDNA samples from infected potato leaves. Samples were collected at 3 days post-infection (dpi) with P13626 and P13527. Primers specific for the RXLR effector gene PITG_16294 were used for amplification. Amplification of the *EF1a* (*Elongation Factor 1α*) cDNA was included as positive control

Plant pathogen effectors with an avirulence activity can trigger hypersensitive cell death when co-expressed *in planta* with their matching immune receptor even in the absence of the pathogen. In an independent set of experiments that aimed at identifying novel *P. infestans* avirulence effectors, we used *Agrobacterium tumefaciens*-mediated transient transformation (agroinfiltration) to express a collection of 240 RXLR effectors in *Solanum venturii* genotype vnt283–1 carrying the *Rpi-vnt1.1* resistance gene [39]. Six candidates consistently triggered a cell death response typical of hypersensitivity (> 70% on a range of 0 (no) -100% (confluent) cell death) (Additional file 1: Table S13). Among these, PITG_16294, one of the three RXLR effectors with an expression polymorphism within EC-1, was the only one to trigger cell death only in genotypes that carry the *Rpi-vnt1.1* gene, including a population of 95 individuals that segregate for this resistance gene (Additional file 1: Table S14). To confirm that PITG_16294 activates *Rpi-vnt1.1*, we co-expressed the two genes in the susceptible potato cv. Désirée (Fig. 3a). We detected hypersensitive cell death only when PITG_16294 is co-expressed with *Rpi-vnt1.1* or the closely related resistance gene *Rpi-vnt1.2* (Fig. 3a). Altogether, these results indicate that PITG_16294 is *Avrvnt1*, the effector that activates *Rpi-vnt1* immunity.

These results prompted us to test whether the two EC-1 isolates show differential virulence on potato plants expressing the *Rpi-vnt1.1* resistance gene. We used the P13527 and P13626 isolates to inoculate transgenic potato cv. Désirée lines expressing *Rpi-vnt1.1*. The two isolates produced strikingly different phenotypes on these plants (Fig. 3b). Whereas P13527 triggered a local immune reaction typical of the hypersensitive response, P13626 infected *Rpi-vnt1.1* potato. Both isolates were virulent on potato cv. Désirée, which is not known to carry any effective resistance gene against *P. infestans*. RT-PCR on the infected plants confirmed the reduced biomass of the avirulent P13527 on *Rpi-vnt1.1* potato and the absence of PITG_16294 transcripts in P13626 (Fig. 3c). We conclude that evasion of *Rpi-vnt1.1*-mediated resistance is probably conferred by absence of PITG_16294 (*Avrvnt1*) transcripts.

## Discussion

Our study revealed the molecular changes underpinning phenotypic plasticity in a pandemic asexual lineage of *P. infestans*. We determined that the EC-1 clonal lineage undergoes significant levels of diversification at the sequence and gene expression level. For the most part, we did not observe the patterns of genome evolution previously described in *P. infestans* possibly because in the present study we examined a shorter evolutionary timescale compared to earlier intra- and inter-specific comparative studies [7, 32, 33]. Therefore, the two-speed genome concept applies only to a limited degree to the relatively recent radiation of the EC-1 asexual lineage. Nonetheless, genes in GSR appeared to exhibit higher levels of structural variation. Linking genome architecture to accelerated adaptive evolution can also be relevant to the micro evolutionary timescales observed in agricultural systems. Field pathogen monitoring must, therefore, probe beyond genotyping and genome sequencing to take into account phenotypic plasticity as exemplified by the observed gene expression polymorphisms. In order to fully capture pathogen evolutionary dynamics, approaches based on RNA-seq, such as the recently developed field pathogenomics method [10, 14] must be used to complement classical population genetics studies.

We observed that the asexual EC-1 lineage can exhibit phenotypic plasticity in the absence of apparent genetic mutations resulting in a dramatic increase in virulence on a potato carrying the *Rpi-vnt1.1* gene. Such variant alleles may be epialleles that arose through epigenetic processes in the underlying genes as observed in other plant pathogens [40–42]. A recent report shows that DNA methylation in the form of N6-methyladenine (6mA) is an epigenetic mark of *P. infestans* that could be linked to adaptive evolution in this pathogen [43]. By contrast, the 5-methylcytosine (5mC) modification has not been detected in *P. infestans* [43, 44]. Furthermore, changes in histone post-translational modifications and chromatin remodelling could play a role in these processes too [44]. However, it is also possible that a genetic trigger, possibly a cis- or trans-structural variant, has resulted in the absence of *Avrvnt1* transcripts. Nonetheless, our observation that on/off expression polymorphisms may predominantly affect effector genes points to a potential adaptive process. In the future, understanding the genetic basis of the processes that underlie expression polymorphism and gene silencing is critical to fully appreciate the evolutionary potential of asexual plant pathogen lineages [45, 46].

## Conclusion

Asexually reproducing organisms are generally thought to be less able to cope with rapid environmental changes due to the lack of genetic recombination. Nonetheless, asexual lineages of fungal and oomycete pathogens can cause lasting disease outbreaks and are known to evolve to evade host immunity when exposed to disease-resistant hosts. We analyzed genetic and gene expression polymorphisms in the *P. infestans* clonal lineage EC-1. EC-1 was first detected in the 1990s and is thought to be a relatively young asexual lineage. Despite this, we documented a notable level of genetic and gene expression polymorphisms. In particular, silencing of the RXLR effector gene *Avrvnt1* enabled virulence on a resistant potato cultivar. Our findings shed light on the mechanisms underlying genome evolution and phenotypic plasticity in an economically important plant pathogen, and highlight the capacity of asexual filamentous pathogens to adapt to rapid environmental changes.

## Methods

### Genome sequencing and analyses

Genomic DNA extraction from mycelia of *P. infestans* isolates P13527, P13626 and 06_3928A, library preparation and Illumina next-generation sequencing reactions were performed as described in [7] using the Illumina Genome Analyzer IIx (Illumina). The expected length of the paired-end reads generated was 76 bp. Sequencing reads with lengths different from 76 bp as well as reads containing Ns were removed with in-house scripts. Filtered Illumina reads were aligned to the reference genome sequence of *P. infestans* T30–4 [32] with the Burrrows-Wheeler Transform Alignment (BWA) software. Average breadth of read coverage, read depth per gene and gene copy numbers were calculated as described in [7]. Genes with no coverage were considered absent. Copy numbers were estimated from the Average Read Depth (ARD) as previously described [7]. ARD for the coding sequence of a gene ‘g’ (ARD [g]) was calculated and adjusted according to GC content. Adjusted ARD for a gene ‘g’ (AARD [g]) belonging to the i^th^ GC content percentile was obtained by the formula AARD [g] = ARD [g] * mARD / mARD_GC_ where mARD is the overall mean depth of all coding sequences and mARD_GC_ is the mean depth for genes in the i^th^ GC content percentile. Copy Number for a gene ‘g’ (CN [g]) was calculated as AARD [g] / mARD. Copy Number Variation for a gene ‘g’ (CNV [g]) was calculated as CN [g] in P13527 – CN [g] in P13626. Genes with the same CN in both isolates will have CNV [g] = 0. Genes with CNV [g] ≥ 1 or CNV [g] ≤ -1 where considered as having copy number polymorphisms between P13527 and P13626. *P. infestans* isolates P13527 and P13626 are from World Oomycete Genetic Resource Collection at UC Riverside, CA as described in [16]. Isolate 06_2938A was obtained from outbreaks of potato late blight across Great Britain (GB) as previously described in [7].

We used the same set of homozygous and heterozygous SNPs in [16]. SNPs were called by comparing each isolate with the T30–4 genome sequence. Allele counts for each position were estimated using pileup from SAMtools [47]. SNPs were called as high quality if depth coverage of reads was > = 10. If a high-quality SNP had been detected in the other strains, SNPs with depth coverage of reads > = 3 were rescued. Allele concordance > = 80% was regarded as homozygous SNPs, and allele concordance between 20 and 80% was regarded as heterozygous SNPs. The details of SNP calling were described in [16]. Synonymous and nonsynonymous (missense and nonsense) SNPs were estimated using snpEff [38].

### RNA sequencing and analyses

Total RNA was extracted from infected potato leaves at 2 days after inoculation using the Plant RNeasy Kit (QIAGEN). Infection methods followed for this work are described below. Two micrograms of total RNA were used for construction of cDNA libraries using the TruSeq RNA Sample Prep Kit (Illumina) according to the manufacturer’s instruction. The libraries were used for paired-end sequencing with 2 × 76 cycles on Illumina Genome Analyzer IIx (Illumina). Adapter sequences were removed from sequencing reads using cutadapt. Sequencing reads containing Ns were discarded, and then unpaired reads were removed using the perl script “cmpfastq_pe” (http://compbio.brc.iop.kcl.ac.uk/software/cmpfastq_pe.php).

Paired sequencing reads were aligned to the T30-4 consensus transcript sequences using Bowtie2 version 2.1.0. To remove potential PCR artifacts, duplicates of paired sequencing reads, which were exactly mapped to the same positions, were removed using MarkDuplicates of Picard version 1.113. Reads mapped to each transcript sequences were counted with “samtools idxstats” of SAMtools version 0.1.19. Count per million (CPM) for each transcript was calculated.

To search for on/off gene expression polymorphisms between the two EC-1 isolates, a threshold value of CPM was defined based on the comparison between RNA-Seq data and microarray data [7] of the 13_A2 isolate 06_3928A from potato leaves 2 days after inoculation. At first, we estimated cumulative distribution of CPM for the transcripts that showed 2-fold induction in planta compared with mycelia in microarray data (Additional file 2: Figure S5). Of these transcripts, 95% had over 18 CPM, and then 18 CPM was set as threshold of expression of genes when the genes showed zero CPM in one of the isolates. This means that when the gene showed zero CPM in one of the isolates and less than 18 CPM in the other, the gene was not regarded as a gene showing on/off expression polymorphisms. For the scatter plot, variance stabilizing transformation of the count was calculated using DESeq [48]. Subtraction from the transformed value corresponding to CPM = 0 from a value of each gene was used as adjusted values of variance stabilizing transformation.

### Enrichment analyses

Gene annotations and locations described in Additional file 1: Table S15 were used to categorise genes showing polymorphisms in the genome and transcriptome analyses. Location of genes in Gene Sparse Regions (GSR), Gene Dense Regions (GDR), in between GSR and GDR, and with location not determined (ND) is based on [33]. Hypergeometric tests were performed for enrichment analysis to detect effector bias and GSR bias in the sets of polymorphic genes (Additional file 3). “phyper” in the R Stats Package was used for the one-tailed hypergeometric tests. Alpha value was set to 0.05. After Bonferroni correction, alpha values for genetic polymorphisms and expression polymorphisms were 0.003 and 0.025, respectively. Considering that genetic and expression polymorphisms are independent biological phenomena and were detected based on different approaches (genome sequencing and RNA sequencing), the multiple corrections were individually applied.

### Validation of expression polymorphisms

To validate expression polymorphisms in RXLR effector genes, detached Désirée potato leaves were drop-inoculated with 50,000 zoospores/mL of *P. infestans* P13527 or P13626. Zoospores were harvested from P13527 or P13626 mycelia grown in Rye Sucrose Agar (RSA) plates for 10–12 days at 18°C by adding cold sterile distilled water and incubating for 2 h, at 4°C. The resulting zoospores’ suspension was diluted to 50,000 zoospores/mL with cold sterile distilled water and 10 μL droplets were used to inoculate detached leaves. Inoculated leaves were kept in closed trays with high moisture under controlled environmental conditions (18°C, 16 h of light and 8 h of dark). Leaf disks harbouring the inoculation site were collected at 1, 2, 3, 4 and 5 days after inoculation. For each time point, disks corresponding to different leaflets from five different plants were pooled and RNA was extracted with the Plant RNeasy Kit (QIAGEN). For RNA and genomic DNA extraction from mycelia, P13527 and P13626 were grown in modified plich medium as described in [7]. Mycelia grown independently in different plates were pooled and used to perform RNA extraction with the Plant RNeasy Kit (QIAGEN) or genomic DNA extraction with the Omniprep Kit (G-Biosciences). For RT-PCR experiments, 5–10 μg of total RNA from infected leaf tissue and from mycelia were subject to DNAse treatment with the TURBO DNA-free Kit (Ambion). cDNA synthesis was performed with 500 ng of DNAse-treated RNA using the SuperScript III First-Strand Synthesis System (Invitrogen) and oligo-dT primers. cDNA (1 μL) and genomic DNA (200 ng) samples were then used as template in PCRs using DreamTaq DNA Polymerase (Thermo Scientific), following manufacturer’s instructions. Primers used were 5’-TAACGACCCCGACCAAGTTA-3′ and 5’-AGAGATGCCAGCCTTTCGTA-3′ for PITG_16294, 5’-AGTGGTGCTCTCGGCGACTCT-3′ and 5’-AGCCCCTCCGTTTCCTGGGT-3′ for PITG_01934, 5’-ATGCGTCTACCGGTACATGTACGT-3′ and 5’-CTAGTCGTAGTTACGCGTCT-3′ for PITG_23131, and 5’-GTCATTGCCACCTACTTC-3′ and 5’-CATCATCTTGTCCTCAGC-3′ for the *Elongation Factor 2 * (*EF2*) control. Presence/absence of amplification products was evaluated in 1–1.5% agarose gels and the GeneRuler 1 kb Plus DNA Ladder (Thermo Scientific) was used to assess amplicon size.

### Effectoromics screen and validation of PITG_16294’s avirulence activity

To identify novel *P. infestans* avirulence effectors a set of 240 RXLR effectors was synthesized at GenScript (New Jersey, USA). Each effector was obtained in pUC57 vector and sub-cloned by Gateway LR cloning into a binary expression vector. The final expression vector was transferred into *Agrobacterium tumefaciens* strain AGL01 and used to perform a screen in *Solanum venturii* genotype vnt283–1 carrying the *Rpi-vnt1.1* resistance gene [39].

Agroinfiltration of RXLR effectors and/or *R* genes was carried out on wild potato species or Désirée potato plants according to Du and Vleeshoouwers [49]. The abaxial side of leaves of four-week-old plants was infiltrated using *A. tumefaciens* cultures with a final OD_600_ = 0.3. Infiltrated leaves were scored 4 days after agroinfiltration to assess the presence of cell death.

### Assessment of differential virulence on potato plants carrying *Rpi-vnt1*

Fully developed leaves of 10-week old *Rpi-vnt1.1* and wild type Désirée plants were detached and infected with 10 μL drops of a 50,000 zoospores/mL suspension of *P. infestans* P13527 or P13626, as described above. Inoculated leaves were incubated for 3 days under controlled environmental conditions (18°C, 16 h of light and 8 h of dark). Disks of 1 cm diameter were cut from the areas showing *P. infestans* infection, and used directly for RNA isolation using TRI Reagent (Sigma-Aldrich), according to manufacturer’s protocol. For each sample, 10 μg of total RNA was treated with DNase I (Thermo Fisher Scientific) and 1 μg of treated RNA was used for first-strand cDNA synthesis with a mix of oligo-dT and random hexamer primers, using SuperScript II First-Strand Synthesis System (Invitrogen). Diluted cDNA (1 μL of a 1,5 dilution) was used for PCR with gene specific primers (F: 5’-CGAAGTTGACGGCTCCTG-3′, R: 5’-GGCTCGCTTGAACAAATCC-3′), and with control primers for *Elongation Factor 1α* (*EF1α*) (F: 5’-GTCATTGCCACCTACTTC-3′, R: 5’-CATCATCTTGTCCTCAGC-3′). Reactions were performed in 25 μl with 30 amplification cycles (annealing temperature 60°C for both primer pairs) and using homemade Taq polymerase. PCR products were visualized on 1% agarose gel.

## Additional files

Additional file 1: This file contains Supplemental **Table S1.** to **S15.** and their legends. (XLSX 1698 kb)

Additional file 2: This file contains Supplemental **Figure S1.** to **S5.** and their legends. (PDF 749 kb)

Additional file 3: One-tailed hypergeometric tests performed for enrichment analyses to assess effector bias and GSR bias in the sets of polymorphic genes. (PDF 242 kb)

## Abbreviations

CNV: Copy number variation
CPM: Counts per million
GDR: Gene-dense regions
GSR: Gene-sparse regions
LOH: Loss of heterozygosity
SNPs: Single nucleotide polymorphisms
