# Supplementary material for: Gene expression polymorphism underpins evasion of host immunity in an asexual lineage of the Irish potato famine pathogen
Source: BMC Evol Biol. 2018 Jul 5;18:93. doi: 10.1186/s12862-018-1201-6 (PMC6032779; doi:10.1186/s12862-018-1201-6)
Supplement: Supplementary file 2 — This file contains Supplemental Figure S1. to S5. and their legends. (PDF 749 kb) [file 12862_2018_1201_MOESM2_ESM.pdf]

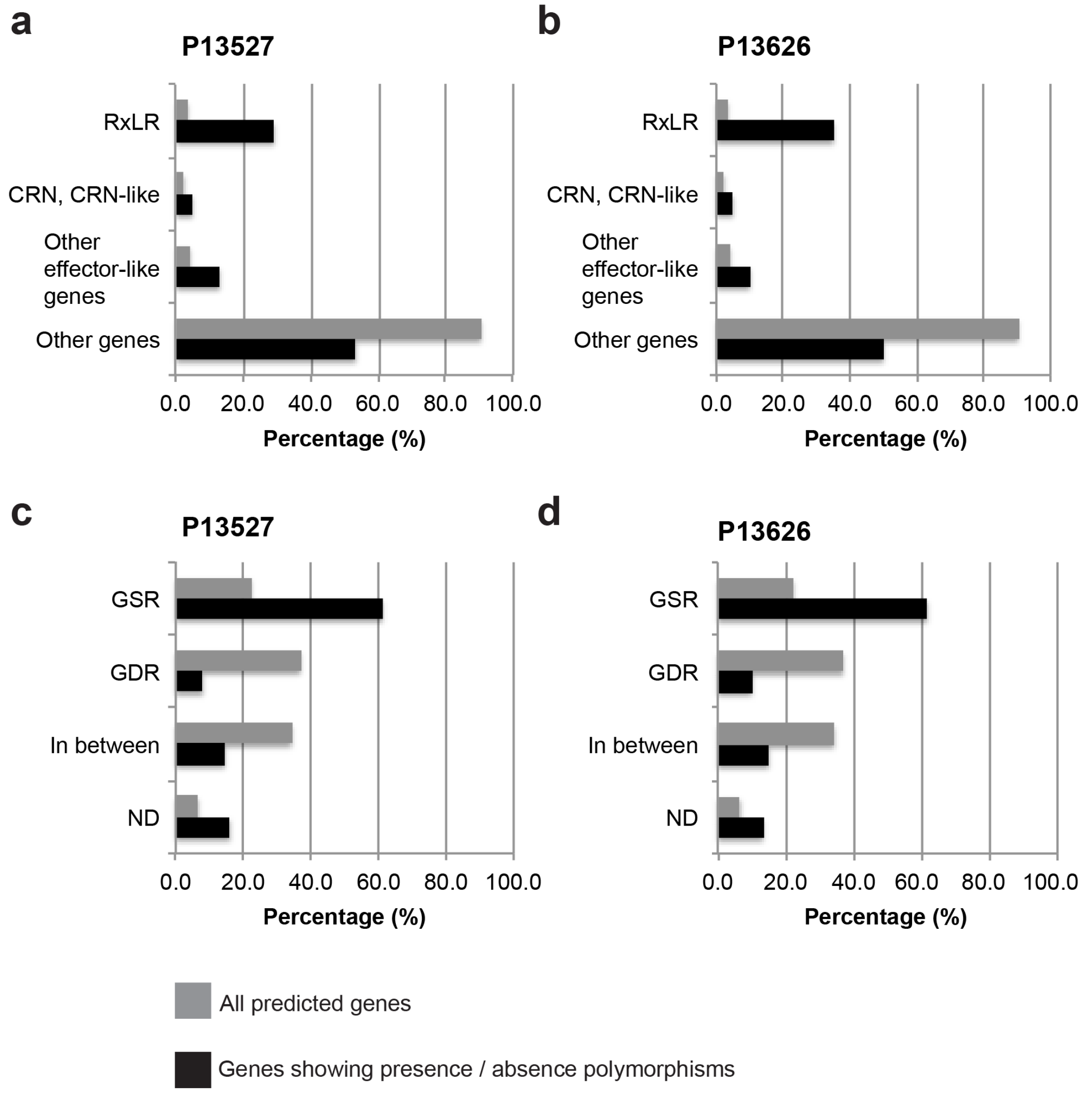

Figure S1: Genes absent in P13527 or P13626 when compared to the reference strain T30-4.

Percentage of RXLR effector genes, Crinkler (CRN) and CRN-like effector genes, other effector-like genes (including genes coding for small cysteine-rich proteins, protein inhibitors, hydrolases, etc.) and non-effector genes in the set of genes absent in P13527 (a) and P13626 (b) when compared to the reference strain T30-4 and in relation to that in all the predicted genes. Percentage of genes located in Gene Sparse Regions (GSR), Gene Dense Regions (GDR), in between GSR and GDR, and with location not determined (ND) in the set of genes absent in P13527 (c) and P13626 (d) when compared to the reference strain T30-4 and in relation to that in all the predicted genes.

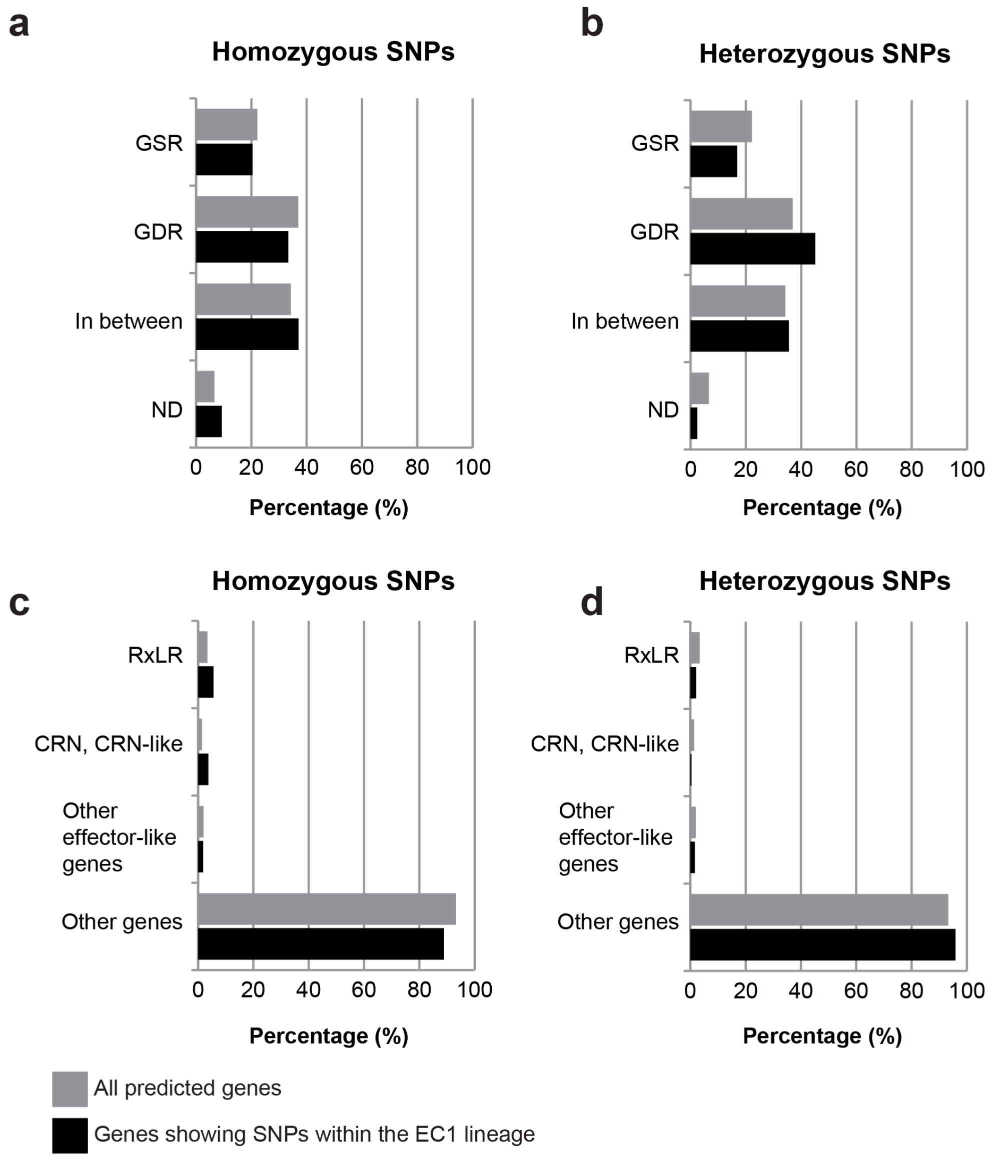

Figure S2: Single nucleotide polymorphisms (SNPs) between P13527 and P13626.

Percentage of RXLR effector genes, Crinkler (CRN) and CRN-like effector genes, other effector-like genes (including genes coding for small cysteine-rich proteins, protein inhibitors, hydrolases, etc.) and non-effector genes in the set of genes harboring homozygous SNPs **(a)** and heterozygous SNPs **(b)** between P13527 and P13626 compared to that in all the predicted genes. Percentage of genes located in Gene Sparse Regions (GSR), Gene Dense Regions (GDR), in between GSR and GDR, and with location not determined (ND) in the set of genes harboring homozygous SNPs **(c)** and heterozygous SNPs **(d)** between P13527 and P13626 compared to that in all the predicted genes.

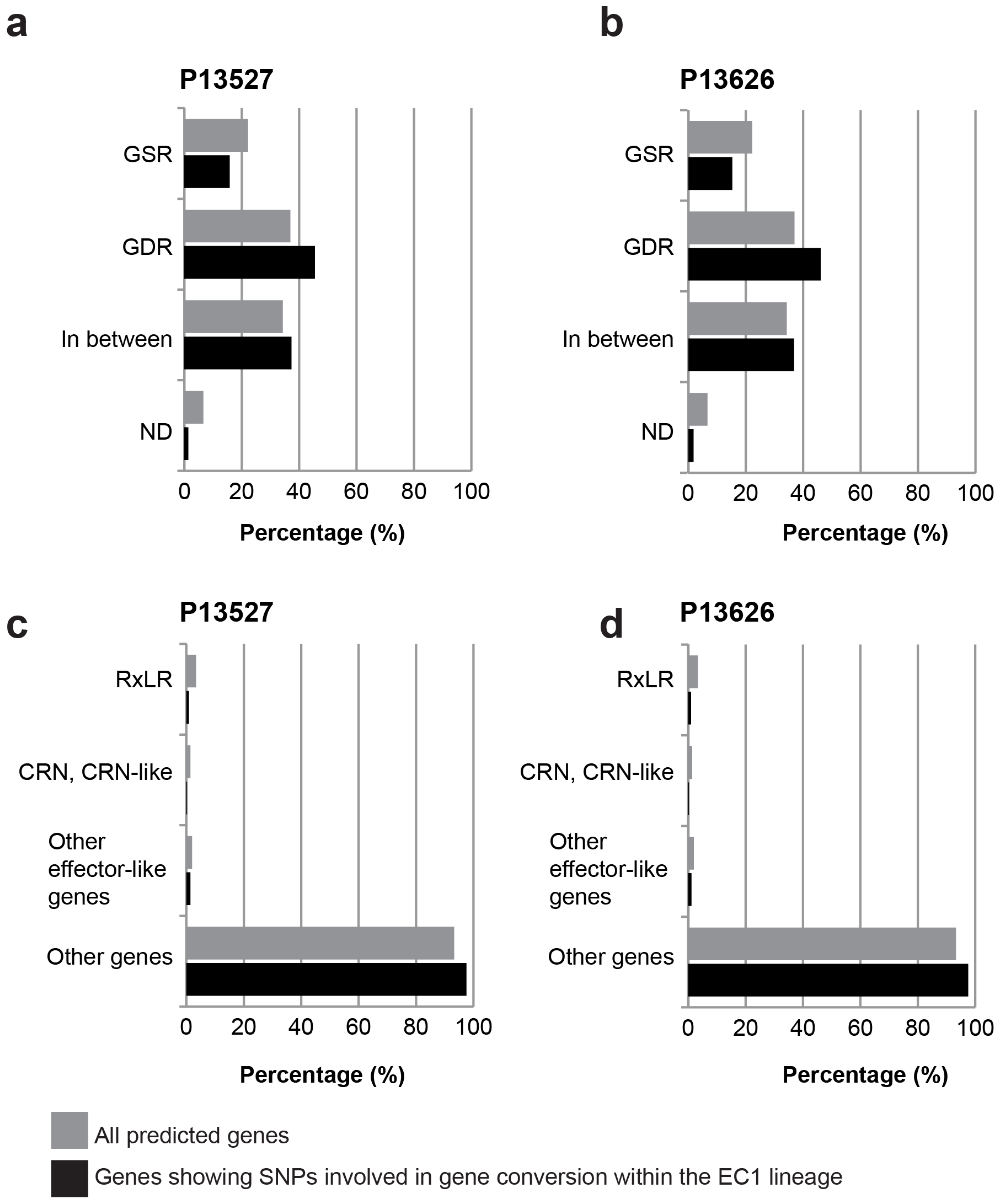

Figure S3: LOH in P13527 and P13626.

Percentage of genes located in Gene Sparse Regions (GSR), Gene Dense Regions (GDR), regions between GSR and GDR (In between), and regions that were not categorized to these three regions (ND) in the set of genes harboring SNPs generated by LOH in P13527 **(a)** and P13626 **(b)**. Percentage of RXLR effector genes, Crinkler (CRN) and CRN-like effector genes, other effector-like genes (including genes coding for small cysteine-rich proteins, protein inhibitors, hydrolases, etc.) and non-effector genes in the set of genes harboring SNPs generated by LOH in P13257 (**c**) and in P13626 **(d)** compared to that in all the predicted genes.

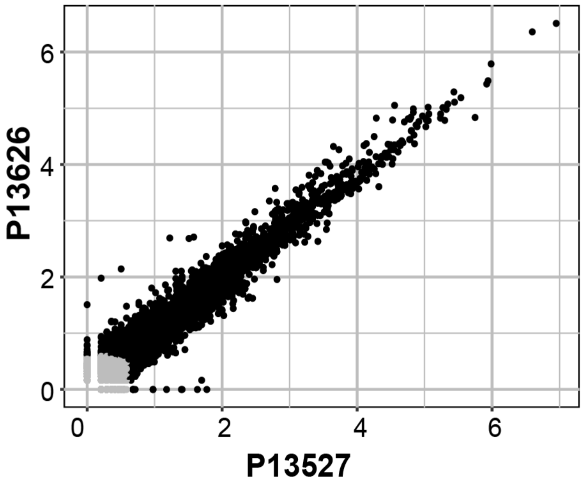

Figure S4: Scatter plot showing overall similarity of gene expression patterns between P13527 and P13627.

Adjusted variance stabilizing transformation of the count data was used for the plot. Pearson correlation coefficient is 0.967. Grey dot indicated genes with CPM value < 18 in both of the isolates.

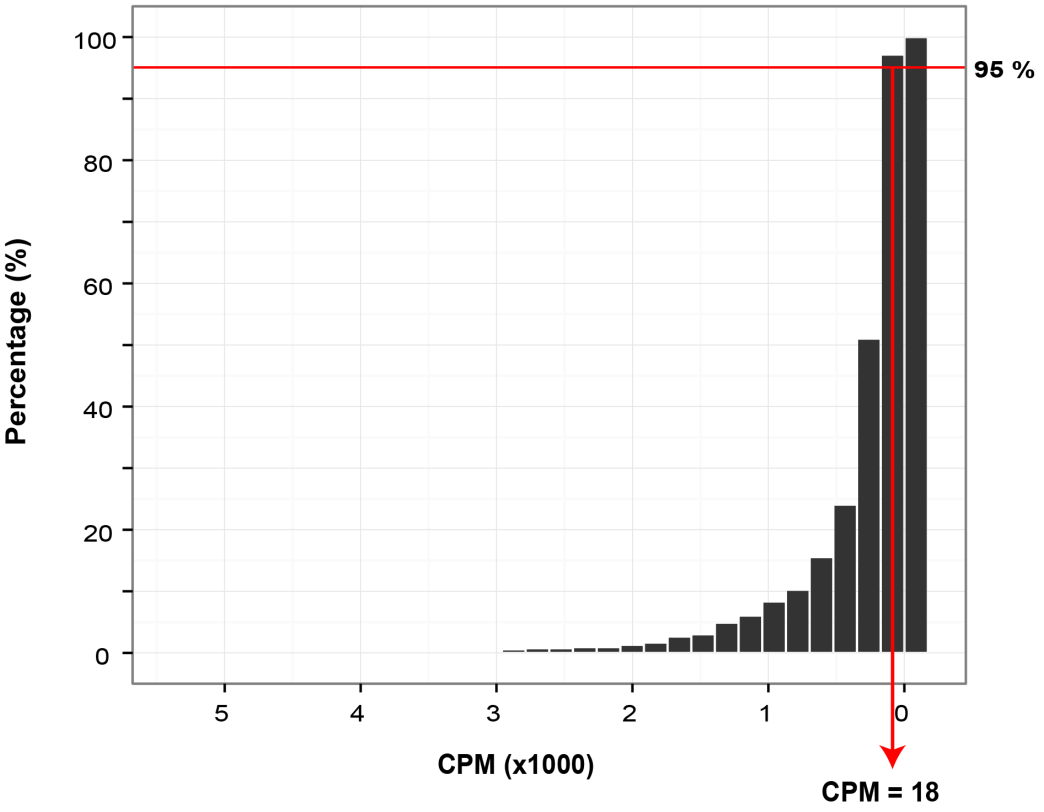

Figure S5: Cumulative Distribution of CPM for the transcripts that showed 2-fold induction in planta compared with mycelia in microarray data.

The arrow indicates the bar including the threshold value of CPM (= 18). Of the transcripts with 2-fold induction in plant, 95% had over 18 CPM.
