# Supplementary material for: Gene expression polymorphism underpins evasion of host immunity in an asexual lineage of the Irish potato famine pathogen
Source: BMC Evol Biol. 2018 Jul 5;18:93. doi: 10.1186/s12862-018-1201-6 (PMC6032779; doi:10.1186/s12862-018-1201-6)
Supplement: Supplementary file 3 — One-tailed hypergeometric tests performed for enrichment analyses to assess effector bias and GSR bias in the sets of polymorphic genes. (PDF 242 kb) [file 12862_2018_1201_MOESM3_ESM.pdf]

<u>SUPPLEMENTARY DATASET 1</u>: One-tailed hypergeometric tests performed for enrichment analyses to assess effector bias and GSR bias in the sets of polymorphic genes.

The sections below list the gene categories, number of genes in each category and scripts used to perform one-tailed hypergeometric tests with "phyper" in the R Stats Package. Results of the enrichment analyses are also included. Alpha value is 0.05. After Bonferroni correction, alpha = 0.05/16 = 0.003 for genetic polymorphisms and alpha = 0.05/2 = 0.25 for expression polymorphisms.

A. ENRICHMENT ANALYSIS FOR GENES DISPLAYING POLYMORPHISMS BETWEEN P13527 AND P13626

1. Gene Presence / Absence Polymorphisms

<u>Hypergeometric test for effector bias</u>

Number of predicted effectors differentially absent	7
Total number of predicted effectors	1752
Total number of predicted non-effectors	16403
Total number of genes differentially absent	12

```
> 1.0-phyper(6,1752,16403,12,lower.tail = TRUE, log.p = FALSE)
[1] 3.940128e-05
```

P(effectors ≥ 7) = $3.9*10^{-5}$

<u>Hypergeometric test for GSR bias</u>

Number of differentially-absent genes in GSR	8
Total number of predicted genes in GSR	4027
Total number of predicted genes in non-GSR	14128
Total number of differentially-absent genes	12

```
> 1.0-phyper(7,4027,14128,12,lower.tail = TRUE, log.p = FALSE)
[1] 0.001205793
```

P(GSR ≥ 8) = 0.0012

2. Gene Copy Number Polymorphisms

<u>Hypergeometric test for effector bias</u>

Number of predicted effectors with copy number variation	10
Total number of predicted effectors	1752
Total number of predicted non-effectors	16403
Total number of genes with copy number variation	69

> 1.0-phyper(9,1752,16403,69,lower.tail = TRUE, log.p = FALSE)
[1] 0.1252412
P(effectors ≥ 10) = 0.1252412

Hypergeometric test for GSR bias

Number of genes with copy number variation in GSR	24
Total number of predicted genes in GSR	4027
Total number of predicted genes in non-GSR	14128
Total number of genes with copy number variation	69

> 1.0-phyper(23,4027,14128,69,lower.tail = TRUE, log.p = FALSE)
[1] 0.01122934

P(GSR ≥ 24) = 0.01122934

3. Gene On/Off Expression Polymorphisms

Hypergeometric test for effector bias

Number of predicted cytoplasmic effectors with expression polymorphisms	4
Total number of predicted cytoplasmic effectors	1016
Total number of other predicted genes	17139
Total number of genes with expression polymorphisms	17

> 1.0-phyper(3,1016,17139,17,lower.tail = TRUE, log.p = FALSE)
[1] 0.01291957

P(cytoplasmic effectors ≥ 4) = 0.0129

Hypergeometric test for GSR bias

Number of genes with expression polymorphisms in GSR	6
Total number of predicted genes in GSR	4027
Total number of predicted genes in non-GSR	14128
Total number of genes with expression polymorphisms	17

> 1.0-phyper(5,4027,14128,17,lower.tail = TRUE, log.p = FALSE)
[1] 0.1554879

P(GSR ≥ 6) = 0.1555

4. Genes with Homozygous SNPs

Hypergeometric test for effector bias

Number of predicted effectors with HomoSNPs	8
Total number of predicted effectors	1752

Total number of predicted non-effectors	16403
Total number of genes with HomoSNPs	54

> 1.0-phyper(7,1752,16403,54,lower.tail = TRUE, log.p = FALSE)
[1] 0.1455074

P(effectors ≥ 8) = 0.0296

<u>Hypergeometric test for GSR bias</u>

Number of genes with HomoSNPs in GSR	11
Total number of predicted genes in GSR	4027
Total number of predicted genes in non-GSR	14128
Total number of genes with HomoSNPs	54

> 1.0-phyper(10,4027,14128,54,lower.tail = TRUE, log.p = FALSE)
[1] 0.6773949

P(GSR ≥ 11) = 0.2305

5. Genes with Heterozygous SNPs

<u>Hypergeometric test for effector bias</u>

Number of predicted effectors with HeteroSNPs	651
Total number of predicted effectors	1752
Total number of predicted non-effectors	16403
Total number of genes with HeteroSNPs	10087

> 1.0-phyper(650,1752,16403,10087,lower.tail = TRUE, log.p = FALSE)
[1] 1

P(effectors ≥ 651) = 1

<u>Hypergeometric test for GSR bias</u>

Number of genes with HeteroSNPs in GSR	1703
Total number of predicted genes in GSR	4027
Total number of predicted genes in non-GSR	14128
Total number of genes with HeteroSNPs	10087

> 1.0-phyper(1702,4027,14128,10877,lower.tail = TRUE, log.p = FALSE)
[1] 1

P(GSR ≥ 1703) = 1

B. ENRICHMENT ANALYSIS FOR GENES DISPLAYING LOSS OF HETEROZYGOCITY (LOH)

1. LOH in P13527

Hypergeometric test for effector bias

Number of predicted effectors with LOH in P13527	121
Total number of predicted effectors	1752
Total number of predicted non-effectors	16403
Total number of genes with LOH in P13527	2370

```
> 1.0-phyper(120,1752,16403,2370,lower.tail = TRUE, log.p = FALSE)
[1] 1
```

P(effectors ≥ 121) = 1

Hypergeometric test for GSR bias

Number of P13527 genes with LOH in GSR	374
Total number of predicted genes in GSR	4027
Total number of predicted genes in non-GSR	14128
Total number of genes with LOH in P13527	2370

```
> 1.0-phyper(373,4027,14128,1996,lower.tail = TRUE, log.p = FALSE)
[1] 0.9999709
```

P(GSR ≥ 374) = 1.0

2. LOH in P13626

Hypergeometric test for effector bias

Number of predicted effectors with LOH in P13626	72
Total number of predicted effectors	1752
Total number of predicted non-effectors	16403
Total number of genes with LOH in P13626	1380

```
> 1.0-phyper(71,1752,16403,1380,lower.tail = TRUE, log.p = FALSE)
[1] 1
```

P(effectors ≥ 72) = 1

Hypergeometric test for GSR bias

Number of P13626 genes with LOH in GSR	211
Total number of predicted genes in GSR	4027
Total number of predicted genes in non-GSR	14128
Total number of genes with LOH in P13626	1380

> 1.0-phyper(210,4027,14128,1380,lower.tail = TRUE, log.p = FALSE)
[1] 1
P(GSR ≥ 211) = 0.99980

C. ENRICHMENT ANALYSIS FOR GENES DISPLAYING PRESENCE / ABSENCE POLYMORPHISMS IN P13527 OR P13626 WHEN COMPARED TO THE REFERENCE STRAIN T30-4

1. *Genes absent in P13527 in relation to T30-4*

Hypergeometric test for effector bias

Number of predicted effectors absent	29
Total number of predicted effectors	1752
Total number of predicted non-effectors	16403
Total number of absent genes	62

> 1.0-phyper(28,1752,16403,62,lower.tail = TRUE, log.p = FALSE)
[1] 4.92939e-14

P(effectors ≥ 29) = 4.9*10^{-14}

Hypergeometric test for GSR bias

Number of genes absent in GSR	38
Total number of predicted genes in GSR	4027
Total number of predicted genes in non-GSR	14128
Total number of absent genes	62

> 1.0-phyper(37,4027,14128,62,lower.tail = TRUE, log.p = FALSE)
[1] 3.653478e-11

P(GSR ≥ 38) = 3.7*10^{-11}

2. *Genes absent in P13626 in relation to T30-4*

Hypergeometric test for effector bias

Number of predicted effectors absent	30
Total number of predicted effectors	1752
Total number of predicted non-effectors	16403
Total number of absent genes	60

> 1.0-phyper(29,1752,16403,60,lower.tail = TRUE, log.p = FALSE)
[1] 1.776357e-15

P(effectors ≥ 30) = 1.8*10^{-15}

Hypergeometric test for GSR bias

Number of genes absent in GSR	37
Total number of predicted genes in GSR	4027
Total number of predicted genes in non-GSR	14128
Total number of absent genes	60

```
> 1.0-phyper(36,4027,14128,60,lower.tail = TRUE, log.p = FALSE)
[1] 5.107959e-11
```

$P(GSR \geq 37) = 5.1*10^{-11}$
